# Photoprotective and Anti-Aging Properties of the Apical Frond Extracts from the Mediterranean Seaweed *Ericaria amentacea*

**DOI:** 10.3390/md21050306

**Published:** 2023-05-18

**Authors:** Serena Mirata, Valentina Asnaghi, Mariachiara Chiantore, Annalisa Salis, Mirko Benvenuti, Gianluca Damonte, Sonia Scarfì

**Affiliations:** 1 Department of Experimental Medicine (DIMES), Biochemistry Section, University of Genova, 16132 Genova, Italy; serena.mirata@edu.unige.it (S.M.); annalisa.salis@unige.it (A.S.); mirko.benvenuti@edu.unige.it (M.B.); gianluca.damonte@unige.it (G.D.); 2 Centro 3R, Interuniversity Center for the Promotion of the Principles of the 3Rs in Teaching and Research, 56122 Pisa, Italy; 3 Department of Earth, Environment and Life Sciences (DISTAV), University of Genova, 16132 Genova, Italy; valentina.asnaghi@unige.it (V.A.); mariachiara.chiantore@unige.it (M.C.); 4 National Biodiversity Future Center (NBFC), 90133 Palermo, Italy

**Keywords:** *Ericaria amentacea*, *Cystoseira amentacea*, antioxidant, anti-collagenase, anti-hyaluronidase, anti-tyrosinase, polyphenols, meroditerpenes, inflammation

## Abstract

There is a growing interest in using brown algal extracts thanks to the bioactive substances they produce for adaptation to the marine benthic environment. We evaluated the anti-aging and photoprotective properties of two types of extracts (50%-ethanol and DMSO) obtained from different portions, i.e., apices and thalli, of the brown seaweed, *Ericaria amentacea*. The apices of this alga, which grow and develop reproductive structures during summer when solar radiation is at its peak, were postulated to be rich in antioxidant compounds. We determined the chemical composition and pharmacological effects of their extracts and compared them to the thallus-derived extracts. All the extracts contained polyphenols, flavonoids and antioxidants and showed significant biological activities. The hydroalcoholic apices extracts demonstrated the highest pharmacological potential, likely due to the higher content of meroditerpene molecular species. They blocked toxicity in UV-exposed HaCaT keratinocytes and L929 fibroblasts and abated the oxidative stress and the production of pro-inflammatory cytokines, typically released after sunburns. Furthermore, the extracts showed anti-tyrosinase and anti-hydrolytic skin enzyme activity, counteracting the collagenase and hyaluronidase degrading activities and possibly slowing down the formation of uneven pigmentation and wrinkles in aging skin. In conclusion, the *E. amentacea* apices derivatives constitute ideal components for counteracting sunburn symptoms and for cosmetic anti-aging lotions.

## 1. Introduction

Macroalgae, the multicellular algae, are large aquatic photosynthetic organisms classified into three classes based on their photosynthetic pigments, the depth in which they live and their ability to absorb sunlight: the green, brown and red algae [1]. The uses of algae in the world go far beyond their simple food consumption; indeed, their anticancer (e.g., Liu et al. [2]), anti-inflammatory and immune system strengthening effects (e.g., Apostolova et al. [3]) have been repeatedly reported. The Mediterranean Sea represents a biodiversity hotspot for the brown algae belonging to the genus *Cystoseira sensu lato* (order Fucales). Chemical investigations of members of this genus led to the discovery of various bioactive secondary metabolites [4,5]. These isolated metabolites are very interesting, both for their biological activity and for their structural complexity. There is also a growing interest in the use of brown algal extracts for their beneficial effects on skin care. Since some of these algae thrive in very shallow waters, they produce bioactive substances that are natural photoprotective agents [6]. These photoprotective substances include amino acids, sulphated polysaccharides, carotenoids and polyphenols. They exhibit a wide range of biological activities, such as the absorption of ultraviolet (UV) rays, inhibition of metalloproteases, and anti-aging, antioxidant and immunomodulatory activities [7]. Indeed, both the oral intake and topical use of brown algae or their extracts demonstrated the ability to reduce the harmful effects of dermal exposure to UV-B rays. It has also been demonstrated that the polyphenols contained in brown algae are able to reduce the proliferation of cancer cells and the volume of UV-B exposure-derived tumors developed in mouse models [8]. Phlorotannins, the phloroglucinol-based polyphenols commonly found in brown algae, appear to be active in the treatment of arthritis [9] and have been reported as effective tyrosinase inhibitors [10] without side effects [11]. Free radicals (i.e., superoxide, nitric oxide and hydroxyl radicals) and other reactive species (i.e., hydrogen peroxide, peroxynitrite and hypochlorous acid) are naturally produced in the human organism, mainly because of aerobic metabolism. They can also be produced due to external factors. Oxidative stress, which often results from the imbalance of the body’s antioxidant status, has been implicated in aging and reactions leading to the generation of reactive oxygen species (ROS) in the skin and is one of the most interesting subjects of cosmetic research because they are involved in various skin diseases and skin aging processes, including atypical pigmentation, wrinkles, thinning, loss of elasticity and last but not least, cancer [12]. Since UV rays promote the generation of ROS in cells, skin aging is usually related to UV exposure [13]. UV rays from the sun, exposure to cigarette smoke and pollutants, and the natural cellular aging process all contribute to the generation of free radicals and ROS. UV rays cause the depletion of cellular antioxidants and antioxidant enzymes (SOD, catalase), initiate DNA damage leading to thymine dimer formation and activate the neuroendocrine system with the release of neuroendocrine mediators. All this leads to the increased synthesis and release of pro-inflammatory mediators from both epidermal and dermal cells [14]. Pro-inflammatory mediators increase capillary permeability, leading to the infiltration and activation of neutrophils and other phagocytic cells. Proteases, such as elastase and metalloproteases, are also released during the inflammatory process, and they are responsible for the degradation of many extracellular matrices (ECM) proteins, which together provide the structural and functional support for the skin tissue, ensuring skin tone, resistance, elasticity and hydration. Therefore, with their hyperactivation, metalloproteases contribute to undermining the skin microarchitecture, favoring premature skin aging [15].

The re-integration of antioxidants in the skin, though exogenous administration, is an extremely valid approach that limits the skin damage induced by ROS caused by UV radiation. These exogenous antioxidants can be both synthetic and natural, and in this second case, the marine environment, thanks to its great biodiversity, can be an important source of these substances to be taken into consideration for the replacement of synthetic antioxidants, which their use is sometimes limited due to the toxic side effects [16,17]. Among all marine organisms, brown macroalgae are an inexhaustible source of these substances, and some of them are, in fact, already used in the field of cosmetics in skin care, sun protection products and anti-aging creams [18].

In this study, we evaluated the anti-inflammatory, photoprotective, antioxidant and hydrolytic enzyme-inhibitory activities on skin cells of two organic extracts obtained from different portions (apices and thalli) of the brown seaweed, *Ericaria amentacea*. Indeed, some bioactive properties of this Mediterranean brown seaweed have already been demonstrated in recent works [19,20,21,22,23,24]. *E. amentacea* is a protected species (listed as “of community interest” according to the European Union’s Habitat Directive 92/43/EEC, under surveillance by the IUCN (International Union for Conservation of Nature), the Berne Convention—Council of Europe, 1979, and the Barcelona Convention—UNEP/MAP, 2009) and its indiscriminate collection, even for pharmaceutical purposes, is undesirable. Although it has been demonstrated that the collection of small apical portions of *E. amentacea* thalli is not harmful to its populations [25], it would be desirable to obtain extracts from laboratory-grown algae or from dedicated seaweed maricultures. Techniques for culturing *E. amentacea* have been refined in recent years [26,27], although obtaining large quantities of biomass from lab cultures is still an issue. Presently, a great deal of effort is dedicated to scaling up laboratory production, mainly with the aim of restoring endangered and damaged natural populations; however, the results of these efforts will also be useful for the future set up of macroalgal mariculture to exploit these precious bioactive molecules. 

The apical component of this brown alga, which grows and develops reproductive structures in late spring/early summer, when solar radiation is maximum, is expected to be particularly rich in antioxidants [28] and, therefore, it is interesting to investigate their beneficial effect on the cells of the epidermis and dermis in comparison to extracts from the rest of the thallus (primary and secondary branches without apices) of the same seaweed. Additionally, 50%-ethanolic (hydroalcoholic) and 100%-dimethyl sulfoxide (DMSO) extracts of the two *E. amentacea* portions were obtained from algae collected in the Ligurian Sea (the Northwestern Mediterranean) in July, and their multifactorial anti-aging properties were studied in two cellular models of keratinocytes and fibroblasts. The final goal was to investigate the potential use of *E. amentacea* derivatives as lenitive and/or anti-aging cosmetics or as antioxidant nutraceutical additives.

## 2. Results and Discussion

### 2.1. TPC and TFC in E. amentacea Extracts

**Scheme 1:**
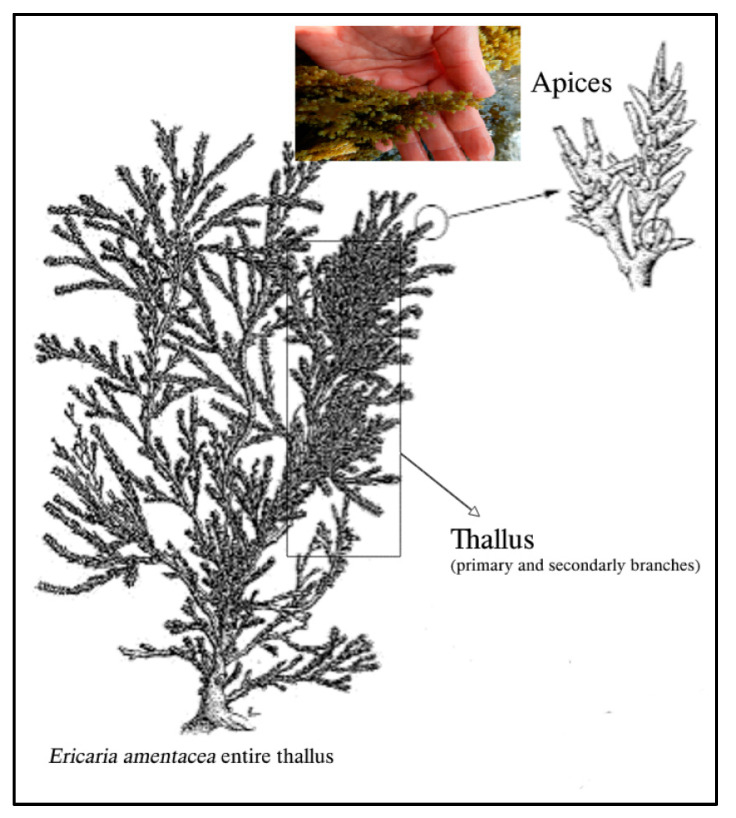
Schematic representation of *E. amentacea* seaweed body parts.

The total phenolic (TPC) and flavonoid (TFC) contents of the extracts obtained from the *E. amentacea* apices and thalli (Scheme 1) using two different solvents, DMSO and a hydroalcoholic solution (50% EtOH), were quantified.

The results in Table 1 show that the EtOH extraction retrieved 2.6-folds higher TPC from the apices of the seaweed compared to the thalli; meanwhile, the DMSO extraction allowed to retrieve 1.4-folds more TPC from the thalli compared to the apices. 

In the various extracts, the TFC percentage, compared to their respective TPCs, was higher in the EtOH samples compared to the DMSO ones. In particular, the TFCs of the EtOH apices and thalli extracts were 11.5% and 12.3% of the respective TPC, while the TFC of the DMSO apices and thalli extracts accounted for 6.1% and 8% of the respective TPC. These results indicate that the apices and the thalli are both rich in polyphenols but qualitatively different since the apices provide high yields of hydroalcoholic soluble polyphenols, while the thalli provide more organic soluble molecular species of the same family. This is entirely possible, however, since polyphenols are a very large and heterogenous family of compounds [29], going from a single phenol moiety (i.e., gallic acid) in the molecule to multiple covalently linked phenol rings (i.e., ellagitannin), which can prefer organic solvent extraction. Therefore, these results could suggest that the fast summer growth of the fronds, and especially of the apices carrying the reproductive structures, may favor a higher synthesis of the hydroalcoholic soluble as compared to the soluble organic species, and then, when the seaweed continues growing, more organic soluble species are produced and are retrievable from the thalli. Similarly, van Alstyne et al. [30] found an intra-thallus distribution of TPC, usually more concentrated in the meristematic zones, i.e., the distal extremities, while Mannino et al. [22] observed the highest TPC values for the *E. amentacea* ethanol extracts in summer when higher protection to solar radiation is needed, in particular, in the most sensitive portions of the thallus (i.e., the newly sprouted branches and the developing reproductive structures). 

The TPC values obtained by the thalli extraction with the two solvents are also in line with the results obtained in our previous paper [23], where *E. amentacea* total fronds collected during the summer season in the Ligurian Sea, with no separation between the apices and thalli, were extracted via the same protocols. Since the apical fronds are only 2–3 cm long compared to the whole thallus (usually more than 10–15 cm), it is likely that the total fronds’ extraction values, obtained in the previous work, were close to those of the thalli obtained in this work and significantly different from the values of the apices of the seaweed. These values are also significantly higher than those obtained by the Atlantic *Cystoseira abies-marina* innovative extraction methods, i.e., by ultrasound or microwave-assisted extraction [31], but are in line with the results from the Mediterranean *Cystoseira compressa* microwave-assisted extraction [32], and from the Mediterranean *Cystoseira sensu lato* species traditional extraction [33]. Both the results from the *Cystoseira compressa* [32] and *Cystoseira sensu lato* [33] analyses notate the seasonal chemical composition differences of the investigated seaweeds, while conversely, our data report, for the first time, the differences in chemical composition and bioactive properties of the extracts from the reproductive portion of the alga compared to the whole yet recently produced thallus, highlighting interesting and promising features.

Furthermore, the EtOH solvent extraction of *E. amentacea* fronds allowed a higher recovery (close to double) of the flavonoid fraction with respect to the DMSO. In this case, no remarkable differences were observed between the extract of the apical fronds and of the thallus with the same solvent, likely indicating, for this class of molecules, a constant synthesis throughout the year, not affected by the seasonal growth of the seaweed nor by the reproductive vs. structural portions of the thallus. This is also an important result, given that flavonoids have demonstrated a protective effect from UV radiation and, consequently, in inhibiting oxidative damage and mitigating skin aging processes [34,35]. Thus, a significant fraction of flavonoids within the extracts make them optimal ingredients for sunburn lotions and anti-aging concoctions.

### 2.2. In Chemico Antioxidant Activity

**Figure 1:**
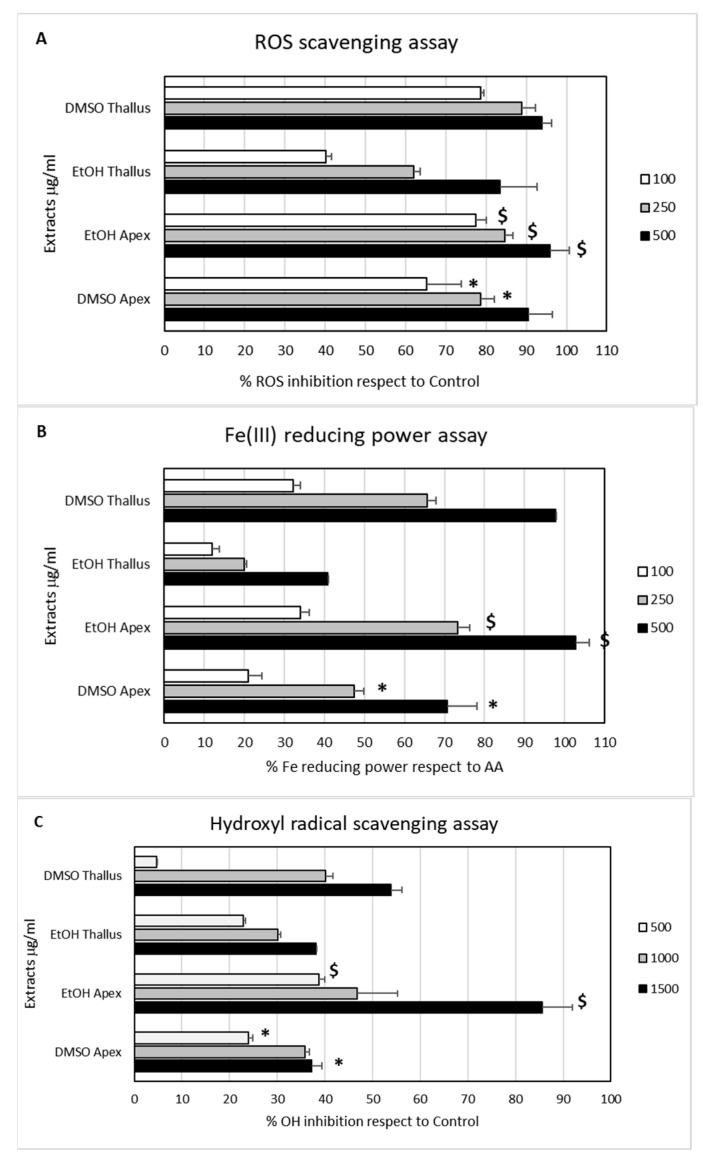

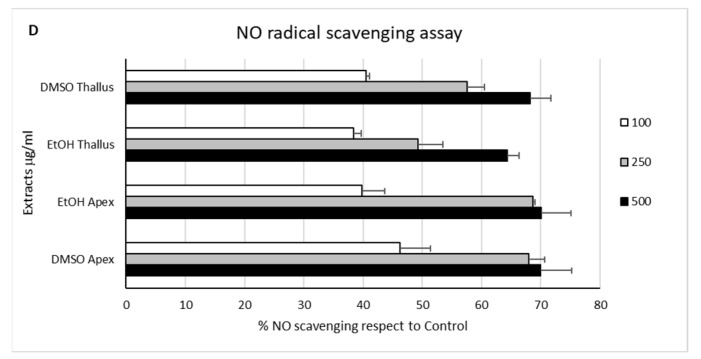
Antioxidant activity of *E. amentacea* extracts in spectrophotometric tests. (**A**) ROS scavenging activity by the DPPH assay. Data are expressed as a percentage of scavenging of the DPPH radical with respect to the absorbance of the DPPH radical alone (control). Symbols indicate significance in paired Tukey test (ANOVA *p* < 0.0001; Tukey of DMSO apex vs. DMSO thallus’ respective concentration, * *p* < 0.05; Tukey of EtOH apex vs. EtOH thallus’ respective concentration, $ *p* < 0.05) (**B**) Fe (III)-reducing power assay, measured by the potassium ferricyanide method. Data are expressed as a percentage of reducing power compared to the Fe reduction of ascorbic acid (AA, concentration 30 μg/mL) positive control. Symbols indicate significance in paired Tukey test (ANOVA *p* < 0.000001; Tukey of DMSO apex vs. DMSO thallus’ respective concentration, * *p* < 0.05; Tukey of EtOH apex vs. EtOH thallus’ respective concentration, $ *p* < 0.005). (**C**) OH radical scavenging activity by Mohr’s salt assay. Data are expressed as a percentage of inhibition of OH radical production with respect to the absorbance of the OH radical-producing solution alone (Control). Symbols indicate significance in paired Tukey (ANOVA *p* < 0.0001; Tukey of DMSO apex vs. DMSO thallus’ respective concentration, * *p* < 0.05; Tukey of EtOH apex vs. EtOH thallus’ respective concentration, $ *p* < 0.05). (**D**) NO radical scavenging activity was measured by the sodium nitroprusside method coupled with the Griess assay. Data are expressed as a percentage of inhibition of NO production with respect to the absorbance of the NO radical-producing solution alone (Control).

The antioxidant properties of the four *E. amentacea* extracts were measured by means of several in chemico assays, namely by measuring the DPPH, hydroxyl and nitric oxide radical scavenging abilities, as well as the Fe-reducing power (Figure 1). 

The DPPH scavenging assay, quantifying the whole ROS scavenging activity (Panel A), provided remarkable dose-dependent antioxidant properties. Based on the inhibition values obtained in the range of the tested concentrations, we extrapolated the DPPH EC75 values of each extract. In particular, we measured EC75 activities of 233.7 and 45.4 μg/mL for the DMSO apices and thalli extracts and 41.9 and 404.1 μg/mL for the EtOH apices and thalli extracts, respectively. These data indicate the retrieval of significant amounts of antioxidant molecular species from both the apices and thalli of the seaweed with both solvents but with some differences, with the highest concentrations obtained by EtOH solvent extraction in the apices and, conversely, by the DMSO solvent extraction in the thalli. This result probably reflects what was already observed for the TPC and TFC of the same extracts. The Fe-reducing power assay (Panel B) revealed a similar dose-dependent behavior of the extracts. The extrapolated EC75 activities for the extracts were 512.5 and 343.1 μg/mL for the DMSO apices and thalli extracts and 311.6 and 970.2 μg/mL for the EtOH apices and thalli extracts, respectively. In a similar way to the DPPH, in the Fe-reducing power assay, the highest activity was found in the EtOH apices extract, followed by the DMSO thalli extract. These results confirm the high antioxidant potential of the four *E. amentacea* extracts that were to be expected from the TPC and TFC analyses of the same extracts reported in Table 1. These are in line with the results from the Mediterranean *C. compressa* extracts [32] and are significantly higher than those obtained from the Atlantic *C. abies-marina* extracts [31]. The lower TPC yields and antioxidant activities of the latter could also be due to the different location of the seaweed, the Atlantic Ocean, with respect to the other *Cystoseira* spp. studies from different locations in the Mediterranean Sea, i.e., the Ligurian Sea in our case and the Adriatic Sea in the Cagalj et al. study [32].

The two final assays were performed to quantify the ability of the extracts to specifically scavenge the biologically relevant hydroxyl (OH) and nitric oxide (NO) radicals. These highly reactive molecules are produced in large quantities in states of cellular oxidative stress and during immune responses as second messengers and defense mechanisms; however, they can seriously damage cell integrity and survival in severe situations [12]. The highest OH radical scavenging activity (Panel C) was again measured for the EtOH apices extract. For this extract, we extrapolated an EC50 of 869.2 μg/mL, followed by the values of the other extracts, which were all above the 1 mg/mL EC50 threshold, with the DMSO thalli extract being 1326.5 μg/mL and, lastly, the DMSO apices and the EtOH thalli having an EC50 of 2097 and 2291.6 μg/mL, respectively. Finally, the potential to scavenge the highly reactive NO radical provided very close EC50-calculated values for all four extracts (Panel D), with the DMSO apices and thalli extracts measuring 163.9 and 207.9 μg/mL, while the EtOH apices and thalli retrieved the values of 234.2 and 272.8 μg/mL, respectively. Overall, these results indicate that the apices of the seaweed in summer possess higher quantities of ROS scavenging molecules and that, in the apices of the fronds growing in summer, i.e., the younger and reproductive parts of the seaweed, *E. amentacea* preferably produces higher quantities of water-soluble antioxidant molecules as compared to the arsenal of molecules with a similar activity present in the already grown thalli. Conversely, both solvent treatments, independently of the section of seaweed extracted (apices or thalli), were able to extract molecules with similar EC50 NO scavenging activities. This is an indication of the production of NO counteracting molecular species with good solubility in both hydroalcoholic and organic solvents, with no significant production increase in the growing apices during the summer season. The various scavenging activities by the extracts shown here also confirm the wide diversity of molecules and secondary metabolites with bioactive properties produced by this brown seaweed, which are already partly reported in the literature [4].

### 2.3. Cell Proliferation and Rescue from Stressful Stimuli

**Figure 2:**
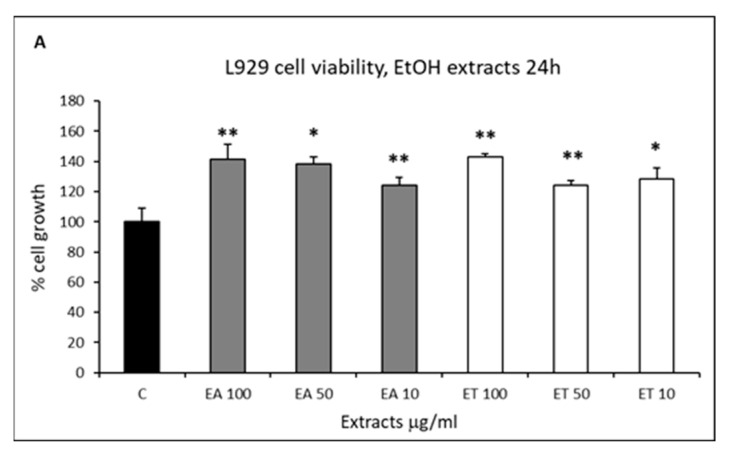

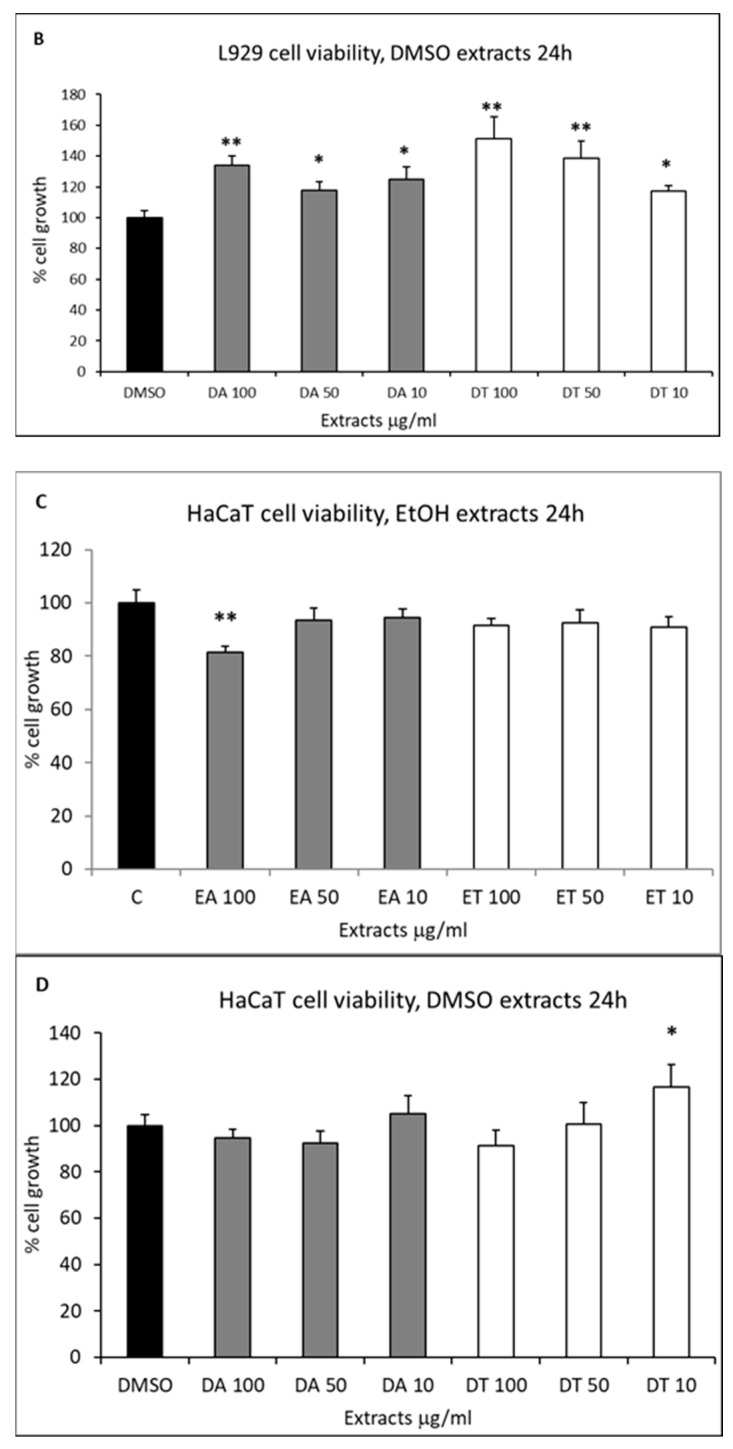
Cell viability evaluation. (**A**,**B**) L929 fibroblast cell growth evaluation by MTT test at 24 h in the presence of increasing concentrations of *E. amentacea* 50%-ethanol (**A**) and DMSO (**B**) extracts—gray bars, apices extracts; white bars, thallus extracts, respectively. Results are expressed as cell percentages with respect to the control. Asterisks indicate significance in paired Tukey test (ANOVA, *p* < 0.00005; Tukey of respective bar vs. C: * *p* < 0.01 and ** *p* < 0.005). (**C**,**D**) HaCaT keratinocyte cell growth evaluation by MTT test at 24 h in the presence of increasing concentrations of *E. amentacea* 50%-ethanol (**A**) and DMSO (**B**) extracts (ANOVA, *p* < 0.000001; Tukey of respective bar vs. C: * *p* < 0.05 and ** *p* < 0.005).

Before assessing the pharmacological and/or cosmetic properties of the four *E. amentacea* extracts in humans, their biocompatibility was analyzed by measuring the cell viability on two skin-derived cell lines, L929 fibroblasts and HaCaT keratinocytes. The results projected a good perspective on their biocompatibility, given that neither of the four extracts showed significant cytotoxicity on both cell lines (Figure 2). 

Conversely, significant cell growth-promoting activity (up to 1.4-folds increase) was revealed upon treatment of the fibroblasts with both the EtOH and DMSO extracts, with no relevant differences between the apices and thalli, especially at the highest concentration tested (Panels A and B, respectively). In the case of human keratinocyte treatment with the extracts, neither significant cytotoxicity nor a remarkable cell growth-promoting effect was observed, except for a slight cell decrease in the highest apices’ EtOH extract concentration and a slight increase in the lowest thalli DMSO concentration (Panels C and D, respectively). These results suggest a safe use of the *E. amentacea* extracts in cosmetic formulations for skincare products, also highlighting a potential dermal thickening effect due to the fibroblast cell growth-promoting effect observed in these experiments. Indeed, with aging, the turnover of epidermal cells decreases, and this explains why the wound healing time increases and desquamation becomes less effective. The dermis also becomes atrophic with a reduced number of fibroblasts [13]. Thus, a product promoting fibroblast proliferation is a really coveted property in cosmetic formulations since the appearance of wrinkles is primarily due to the insufficient proliferation and ECM deposition of these cells in the dermal layer. These results are surprisingly different from others reported in the literature on the cytotoxic and antitumor activity of different *Cystoseira* spp. extracts, i.e., those of *C. tamariscifolia* [36] and *C. barbata* [37], to cite a few. Although, in many cases, the extracts were obtained in more aggressive conditions (heating) by use of strong organic solvents, such as hexane and diethyl ether for *C. tamariscifolia* and acetone for *C. barbata*, respectively. Thus, even if we cannot exclude the presence of cytotoxic/antitumor secondary metabolites in *E. amentacea* seaweed, we can confirm that the molecular species retrieved by the extraction procedures used in this paper, i.e., DMSO and hydroalcoholic, can be considered safe for human use.

**Figure 3:**
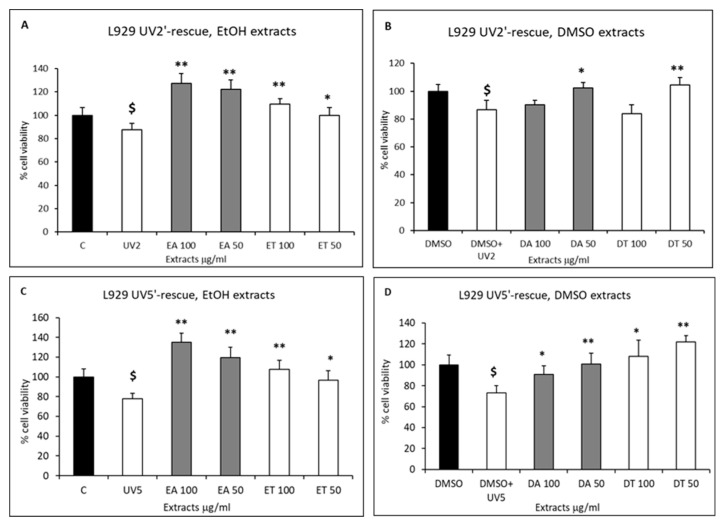

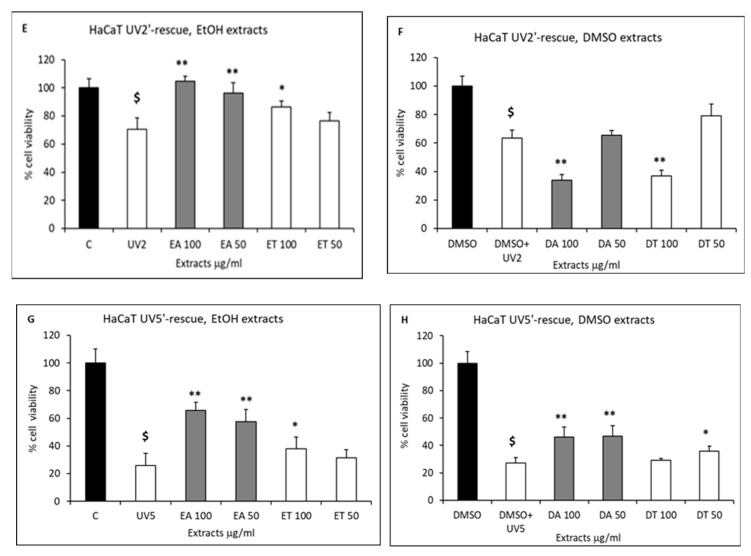
Cell death rescue evaluation after UV challenge. (**A**–**D**) Cell death rescue from 2 min UV (**A**,**B**) and 5 min UV (**C**,**D**) challenge of L929 fibroblasts in the presence of 50%-ethanol and DMSO extracts, evaluated by MTT test 24 h after the stimulus. Gray bars, apices extracts; white bars, thallus extracts, respectively. Results are expressed as a cell percentage with respect to control, untreated cells. Symbols indicate significance in paired Tukey test (ANOVA, *p* < 0.0005 in all panels; Tukey of UV2 or UV5 vs. C $ *p* < 0.005; Tukey of respective bar vs. UV2: * *p* < 0.05 and ** *p* < 0.001; Tukey of respective bar vs. UV5: * *p* < 0.05 and ** *p* < 0.0005). (**E**–**H**) Cell death rescue from 2 min UV (**E**,**F**) and 5 min UV (**G**,**H**) challenge of HaCaT keratinocytes in the presence of 50%-ethanol and DMSO extracts, evaluated by MTT test 24 h after the stimulus (ANOVA, *p* < 0.000001 in all panels; Tukey of UV2 or UV5 vs. C $ *p* < 0.0001; Tukey of respective bar vs. UV2: * *p* < 0.01 and ** *p* < 0.0005; Tukey of respective bar vs. UV5: * *p* < 0.05 and ** *p* < 0.005).

**Figure 4:**
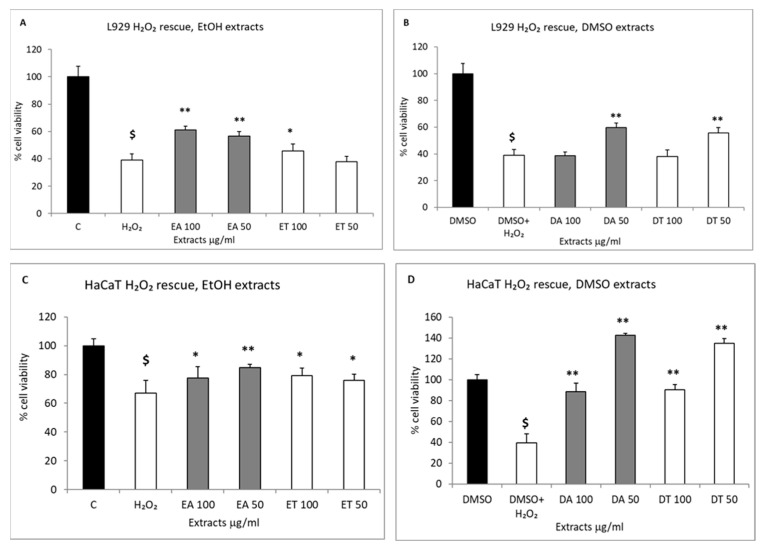
Cell death rescue evaluation after H_2_O_2_-challenge. (**A**,**B**) Cell death rescue from 200 μM H_2_O_2_-challenge of L929 fibroblasts in the presence of 50%-ethanol and DMSO extracts, evaluated by MTT test 24 h after the stimulus. Gray bars, apices extracts; white bars, thallus extracts, respectively. Results are expressed as cell percentages with respect to control, untreated cells. Symbols indicate significance in paired Tukey test (ANOVA, *p* < 0.000001; Tukey of H_2_O_2_ vs. C, $ *p* < 0.0001; Tukey of respective bar vs. H_2_O_2_: * *p* < 0.05 and ** *p* < 0.001). (**C**,**D**) Cell death rescue from 500 μM H_2_O_2_-challenge of HaCaT keratinocytes in the presence of 50%-ethanol and DMSO extracts, evaluated by MTT test 24 h after the stimulus (ANOVA, *p* < 0.0005; Tukey of H_2_O_2_ vs. C, $ *p* < 0.0005; Tukey of respective bar vs. H_2_O_2_: * *p* < 0.05 and ** *p* < 0.0005).

The extract’s beneficial properties on skin cell survival in stressful conditions were also tested. Fibroblasts and keratinocytes were challenged with UV-B to mimic the sun’s dangerous radiation effects and with hydrogen peroxide to mimic cell damage by oxidative stress (Figure 3 and Figure 4, respectively). 

The results show that in both cases, the extracts were able to rescue, partially or totally, the detrimental effects of the two stressful stimuli. In detail, UV-B radiation for 2 or 5 min induced, respectively, 13% or 26% cell death in the L929 fibroblasts compared to the control cells (Figure 3A,B and C,D, respectively).

The apices and thalli EtOH extracts were able to nullify the induced cell death, both in the UV 2′-exposure (Panel A) and the 5′-exposure (Panel C) at both concentrations tested (50 and 100 μg/mL). In addition, the apices extract (gray bars) showed cell growth values significantly exceeding those of the control—the untreated cells—in a dose-dependent way. Conversely, the apices and thalli DMSO extracts were more effective at the lowest concentration tested, which, also in this case, counteracted the UV-induced cell death both after the 2′ and 5′ radiation (Panels B and D, respectively). No significant differences were observed between the apices and thalli DMSO extract rescuing effects. UV-B radiation in the HaCaT keratinocytes led to higher cell death percentages as compared to the fibroblasts and amounted to 30% and 75% for the 2′ and 5′ exposures, respectively (Figure 3E,F and G,H). In the case of the EtOH extracts, only the apices (gray bars) were able to effectively counteract the UV-induced toxicity by completely restoring the cell viability in the 2′ UV exposure (Panel E) and partially in the 5′ UV exposure (+40% cell rescue, Panel G). The DMSO extracts exerted a different effect on the UV-exposed keratinocytes; none of them were able to rescue the 2′ UV-induced cell death (Panel F), and only the apices extract (gray bars) was able to partially counteract the 5′ UV-induced cell death (+20% cell rescue, Panel H). 

As far as the hydrogen peroxide-induced cell death (60%) in the L929 fibroblasts (Figure 4A,B) was concerned, the cell damage was partially rescued by the EtOH apices extract in a dose-dependent way (Panel A, gray bars), and by both the apices and thalli DMSO extracts at the lowest concentration tested (Panel B, gray and white bars, respectively). 

Conversely, HaCaT keratinocyte cell death, induced by hydrogen peroxide, was partially rescued by both apices and thalli EtOH extracts (Panel C) and completely by both apices and thalli DMSO extracts (Panel D) at both concentrations tested. These results indicate a prevalent rescuing effect of the apices extracts compared to the thalli, although also in the latter, a significant retrieval of molecules with a beneficial effect is clearly obtained by the two extracting solvents, as indicated by the higher survival rates when compared to the UV- and H_2_O_2_-treated positive controls. These results demonstrate that the growth-promoting effects of the extracts also expand to stressful conditions of the skin, such as in the case of UV exposure and oxidative stress, enhancing the chances of survival of the damaged epidermal and dermal cells and thus slowing down the skin aging process. The percentages of cell survival upon UV radiation and after *E. amentacea* extract administration demonstrate significant and possibly preventive effects, even higher than those observed on the same cellular model (fibroblast cells) with the use of purified phlorotannins from other brown algae administered at μmolar concentrations (from 10 to 250 μM), as reported by Heo et al. [38]. In our case, the single bioactive molecules that are present in the extracts likely have significantly lower concentrations than those used by Heo et al. since, in the cellular setting, they are always used at a 100 μg/mL final concentration. Thus, our results probably show an additive and/or synergic effect of the heterogenous antioxidant and vitaminic compounds exerting such a dramatic effect. This points out the possibility that the polyphenol and meroditerpenes concoctions, as those present in our extracts (see Section 2.7 and De La Fuente et al. [23]), at lower concentrations, perform better than the single-molecule concentrated solutions in counteracting UV damage in skin cells, probably acting at different cellular levels instead of targeting single chemical pathways. However, we must remark that our experimental conditions only demonstrate a preventive action of the extracts on the UV stressful stimulus at the cellular level. In fact, since the extracts were administered to cells before UV exposure, thus mimicking the application of sunscreen, the rescue results likely reflect the sum of both the extracellular UV direct screening effect and the increase in survival due to an intracellular rescuing activity. Further experiments will help us to elucidate the contribution of each effect to the increased cell survival, for example, by the administration of the extracts after UV exposure, which would enable us to discriminate only the intracellular effect on cell survival.

### 2.4. Intracellular Antioxidant Activity

**Figure 5:**
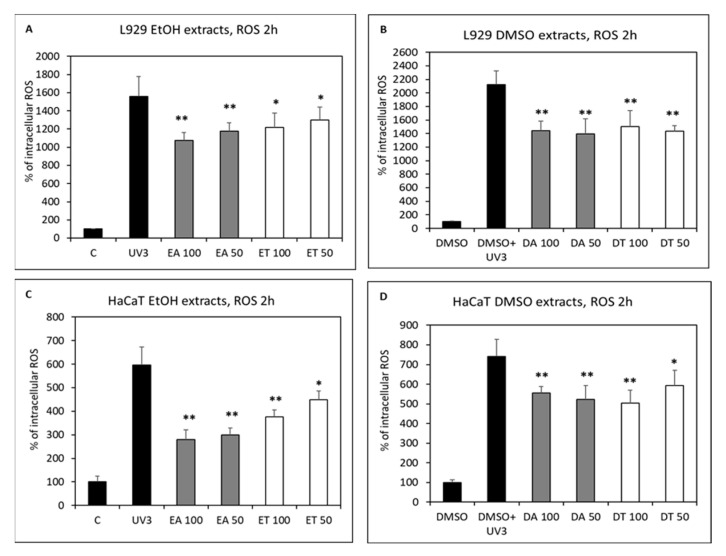

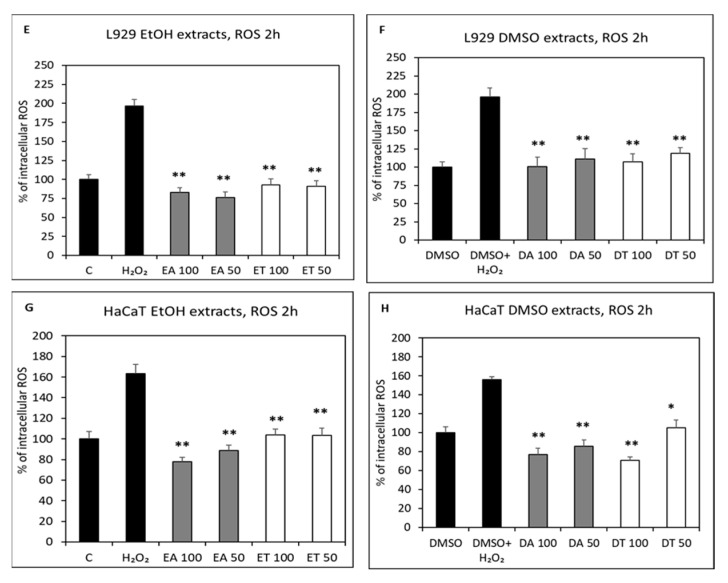
ROS scavenging activity in cellular assays. (**A**,**B**) Intracellular ROS production, measured by DCF fluorometric analysis in L929 fibroblasts incubated for 2 h in the presence or absence of 50 and 100 μg/mL of 50%-ethanol (**A**) or DMSO (**B**) extracts, respectively, after a 3 min UV challenge. Gray bars, apices extracts; white bars, thallus extracts, respectively. Results are expressed as a percentage of ROS production with respect to control, untreated cells. Asterisks indicate significance in the Tukey test (ANOVA *p* < 0.000001; Tukey of respective bar vs. UV3, * *p* < 0.05 and ** *p* < 0.005). (**C**,**D**) Intracellular ROS production in HaCaT keratinocytes incubated with 50 and 100 μg/mL of 50%-ethanol (**C**) or DMSO (**D**) extracts after a 3 min UV challenge (ANOVA *p* < 0.000001; Tukey of respective bar vs. UV3, * *p* < 0.05 and ** *p* < 0.005). (**E**,**F**) Intracellular ROS production in L929 fibroblasts incubated for 2 h with 200 μM H_2_O_2_ in the presence or absence of 50 and 100 μg/mL of 50%-ethanol (**E**) or DMSO (**F**) extracts (ANOVA *p* < 0.000001; Tukey of respective bar vs. H_2_O_2_, ** *p* < 0.0001). (**G**,**H**) Intracellular ROS production in HaCaT keratinocytes incubated for 2 h with 200 μM H_2_O_2_ with 50 and 100 μg/mL of 50%-ethanol (**G**) or DMSO (**H**) extracts (ANOVA *p* < 0.000001; Tukey of respective bar vs. H_2_O_2_, * *p* < 0.001 and ** *p* < 0.0001).

It is well known that most of the cell-damaging effects of UV radiation and acute inflammation are due to the production of high amounts of ROS [12], with the consequent oxidation and deterioration of fundamental cellular components (proteins, nucleic acids, lipids). Therefore, we evaluated the four extracts’ ability to scavenge UV- and H_2_O_2_-derived ROS production (Figure 5), which would explain the cell death-reversing effect of the extracts described in the previous paragraph. 

Indeed, the UV-B-stimulated fibroblasts and keratinocytes (Panels A–B and C–D, respectively) produced remarkable amounts of ROS after a 3 min exposure, as compared to the control cells (from a 15- to 21-fold increase in fibroblasts, Panels A–B, and from a 6- to 7-fold increase in keratinocytes, Panels C–D, respectively). In fibroblasts, the apices EtOH extracts showed a higher percentage of ROS inhibition with respect to the thalli (Panel A, 31% vs. 22% scavenging effect, respectively), while a similar rate of inhibition was observed for the two DMSO extracts (Panel B, ~30%). In the HaCaT keratinocytes, the EtOH extracts from the apices showed the highest rate of ROS inhibition (Panel C, gray bars, 64%) compared to the EtOH extracts from the thalli (Panel C, white bars, 38%) and to both the apices and thalli DMSO extracts (Panel D, 30–32% ROS lowering). Considering the extremely high levels of ROS produced by UV radiation in both cell types, the intracellular scavenging results obtained by the *E. amentacea* extracts, especially the EtOH apices, are indeed significant. These experiments clearly demonstrate the extreme degree of damage that UV radiation can cause by ROS production at the cellular level and the possibility of rapidly assessing the photoprotective activity of compounds in a simple in vitro setting on skin cells, as described here. 

The H_2_O_2_-challenged cells showed a lower intracellular ROS stimulation compared to the UV-B exposure (with a 2-fold increase in fibroblasts, Panels E–F, and a 1.6-fold increase in keratinocytes, Panels G–H, respectively). In both cell types, all four extracts were able to inhibit the ROS production completely; in particular, in the case of the EtOH extract from the apices, the values were even below those of the control cells in both fibroblasts and keratinocytes (Panels E and G, gray bars). Furthermore, in the HaCaT cells, the two DMSO extracts were also able to reduce the H_2_O_2_-induced ROS production below the values of the control cells (Panel H). These data clearly indicate the potent antioxidant and photoprotective activity of the four *E. amentacea* extracts on skin cells, with the hydroalcoholic apices extract again showing the highest pharmacological effect. Moreover, in this case, as already observed in the cell death rescue experiments, the beneficial, antioxidant effect of the extracts is higher than that observed using single purified molecules, as in the case of phlorotannins [38] in a very similar cellular setting, indicating an additive/synergistic effect of the compounds contained in the *E. amentacea* extracts (see Section 2.7). The strong antioxidant activity of the extracts is likely the main mechanism that allows cell survival and growth in the fibroblast and keratinocyte cells after UV and hydrogen peroxide noxious stimuli administration.

### 2.5. Skin Hydrolytic Enzyme Inhibition

**Figure 6:**
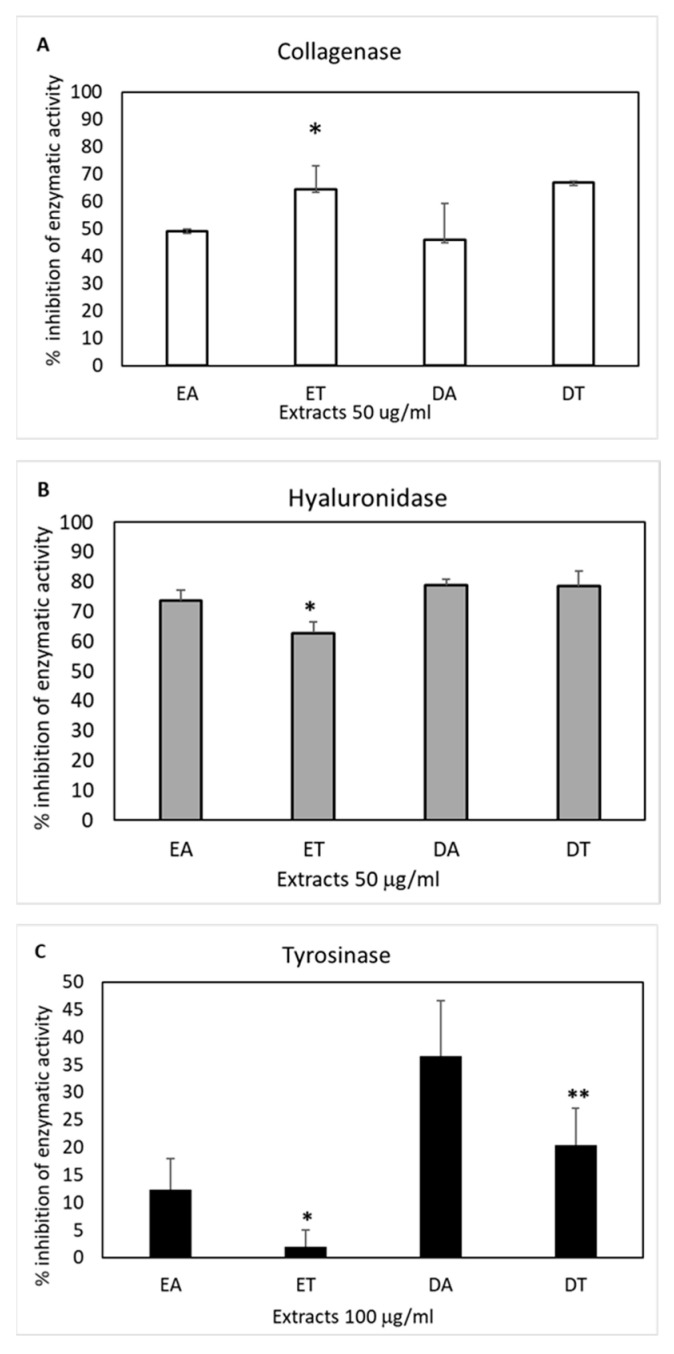
Counteraction of skin hydrolytic enzyme activity. (**A**) Inhibition of collagenase (from *C. histolyticum*) by enzymatic kinetic assay, measured as a reduced degradation of FALGPA, a synthetic peptide that mimics the collagen structure, in the presence of 50 μg/mL of algal extracts as compared to the activity of the control enzyme (in the presence of the respective vehicle solvents). EA and ET, apices and thallus 50%-ethanol extracts; DA and DT, apices and thallus DMSO extracts, respectively. Asterisks indicate significance in paired Tukey test (ANOVA *p* < 0.0005, Tukey of ET vs. EA, * *p* < 0.05). (**B**) Inhibition of hyaluronidase (from bovine testes) by enzyme kinetic assay measured as a reduced degradation of hyaluronic acid in the presence of 50 μg/mL algal extracts as compared to the control enzyme activity (in the presence of the respective vehicle solvents). Asterisks indicate significance in paired Tukey test (ANOVA *p* < 0.0005, Tukey of ET vs. EA, * *p* < 0.05). (**C**) Inhibition of mushroom tyrosinase by the relative enzyme kinetic assay, measured as a reduced conversion of L-Dopa to Dopachrome in the presence of 100 μg/mL of algal extracts as compared to the activity of the control enzyme (in the presence of the respective vehicle solvents). Asterisks indicate significance in paired Tukey test (ANOVA *p* < 0.0005, Tukey of ET vs. EA, * *p* < 0.05; Tukey of DT vs. DA, ** *p* < 0.01).

Skin aging is characterized by the loss of dermal thickness and hydration, wrinkle formation and dyspigmentation with the appearance of unsightly sunspots due to uneven melanin production by melanocytes [13]. These features are mainly due to photo-induced oxidative stress [14], reduced fibroblast proliferation and an imbalance between the synthesis and degradation of important ECM components, such as collagen and hyaluronic acid. Both the number and diameter of collagen fibers are reduced, and the ratio between type III and type I collagen is increased [13]. The approaches to restore dermal thickness span from a daily intake of marine collagen hydrolysate nutraceuticals [39] to hyaluronic acid subcutaneous injections [40] and/or the application of collagen- and/or hyaluronic acid-based cosmetic creams [41,42] and skincare products containing oxidative stress-counteracting vitamins, such as vitamin A-derived retinol, vitamin E and C [43]. The latter has an important double role in skin homeostasis since it is also directly involved in the synthesis and deposition of collagen [44]. An alternative approach to improve dermal thickness is based on the use of the enzymatic inhibitors of collagenases and hyaluronidases to increase the ECM components’ stability [45]. Thus, the four *E. amentacea* extracts were tested for possible anti-collagenase and anti-hyaluronidase activities in spectrophotometric enzymatic tests. The results shown in Figure 6 highlight the presence of molecules with such activities in almost all extracts, although with differences in their efficacy. 

A 45–50% inhibition of collagenase activity (Panel A) was obtained in the presence of the two apices extracts, while a slightly higher effect was observed with the two thalli extracts (65% inhibition). No significant differences were observable between the two extraction solvents (EtOH and DMSO), indicating a collagenase inhibitory activity with promiscuous solubility that is more concentrated in the older part of the seaweed fronds. These results show higher anti-collagenase properties for the *E. amentacea* extracts compared to other seaweeds of the same family, as, for instance, *Cystoseira abies-marina*, whose extracts were obtained by ultrasound or microwave-assisted technologies and never exceeded a 50% enzymatic inhibitory effect [31].

The highest inhibition of enzymatic activity was obtained against the hyaluronidase (Panel B), reaching values close to 80% by the two DMSO extracts and was slightly lower in the presence of the two EtOH extracts, again indicating the presence of inhibitory molecules with promiscuous solubility and no substantial differences between the concentration of the activity in the apices as compared to the thalli. These results show a high hyaluronidase inhibitory potential of the *E. amentacea* extracts, probably due to an additive effect of the various molecular species present in the seaweed. It is known that phlorotannins can exert this effect [46], as demonstrated by the purified molecules from four different algae belonging to the same order of *E. amentacea*, i.e., Fucales; however, their effective concentration is in the order of more than 1 mg/mL of purified molecules. Since, in our case, the highest inhibitory effect was obtained with 50 μg/mL of extracts, we can conclude that probably other types of polyphenols are contributing to the anti-hyaluronidase effect of *E. amentacea*. In addition, the inhibition of collagenase, also known as MMP1, and hyaluronidase can be considered an important antitumor activity since these enzymes are usually overexpressed in spreading cancers, favoring tumor cell proliferation and migration by degrading connective tissue ECM components [46,47,48].

The anti-tyrosinase activity was also tested, given that this enzyme was responsible for the uneven production of melanin from the tyrosine precursor in sunspots. Not all UV rays can affect our skin; UV-C, in fact, is filtered by the ozone layer. UV-A and UV-B, on the other hand, are not completely filtered and, therefore, can cause phenomena such as tanning, wrinkles and hyperpigmentation once absorbed by the layers of the skin. Hyperpigmentation is usually a harmless form of skin pigmentation in which the skin patches become darker than the surrounding normal skin due to the overproduction and accumulation of melanin. Melanogenesis is controlled by the enzyme tyrosinase, a glycoprotein located in the membrane of melanocytes that catalyzes the conversion of L-tyrosine into melanin [49]. A different efficacy was observed in the four extracts in terms of their ability to inhibit the tyrosinase enzyme (Panel C). In particular, at 100 μg/mL, the DMSO extract of the apices showed the highest percentage of inhibition (36%), followed by the thalli DMSO extract (20% inhibition). As far as the EtOH extracts are concerned, while the apices still showed a slight inhibitory activity (12%), the thalli were practically ineffective. Therefore, this enzymatic inhibitory activity seems to be more concentrated in the apices of the seaweed and more compatible with organic solvents. It has been reported that polyphenols belonging to the phlorotannin group show very high anti-tyrosinase activity [6]. As compared with the literature, *E. amentacea* does not seem to possess better properties in this specific activity, compared to other brown algae extracts, i.e., *C. abies-marina* and *E. stolonifera*, showing more significant whitening performances [31,50].

### 2.6. Skin Anti-Inflammatory Effect

**Figure 7:**
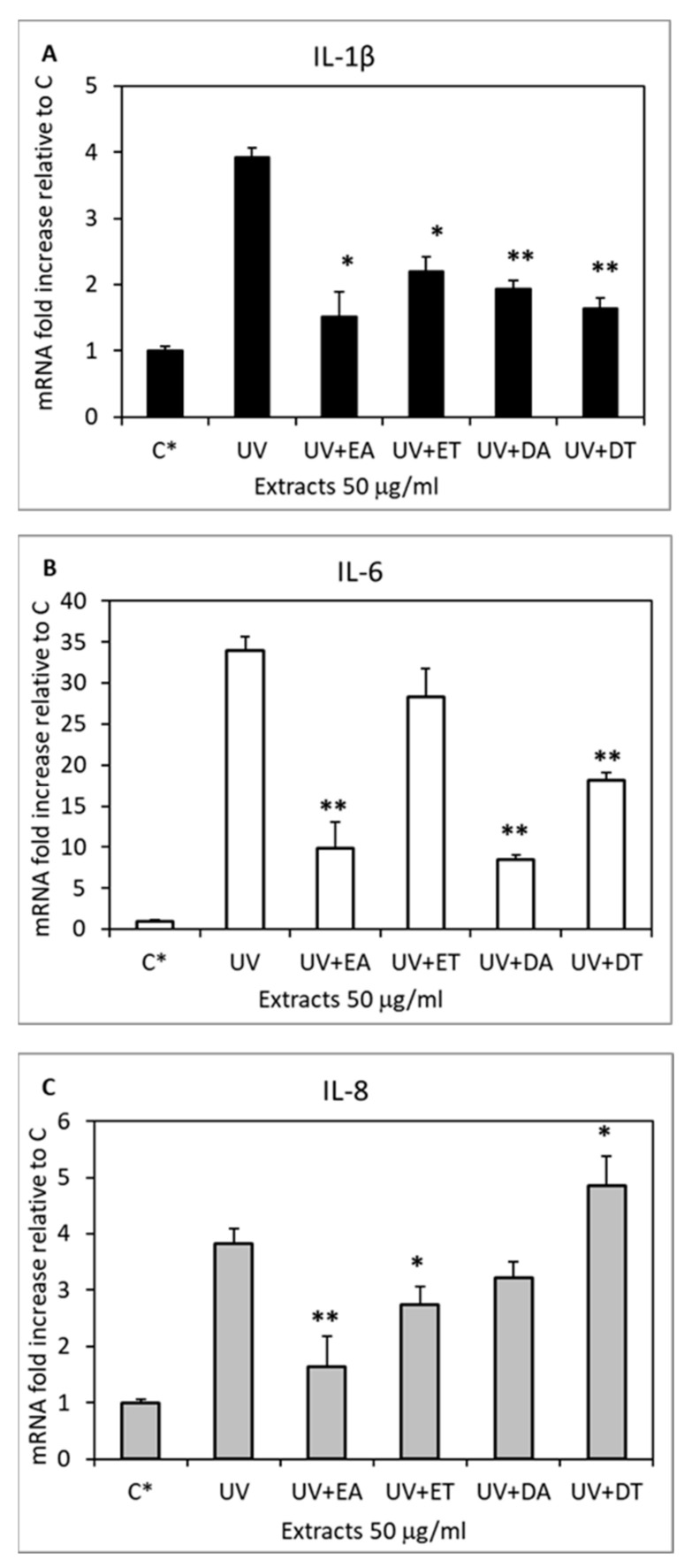
Inhibition of gene expression in UV-challenged HaCaT human keratinocytes. Gene expression was measured by qPCR analyses of IL-1β (**A**), IL-6 (**B**), and IL-8 (**C**) at 6 h, after a 3 min UV challenge and in the presence of 50 μg/mL algal extracts. EA and ET, apices and thallus 50%-ethanol extracts; DA and DT, apices and thallus DMSO extracts, respectively. Data are normalized on the HPRT-1 housekeeping gene and expressed as mRNA-fold increase compared to control cells. Asterisks indicate significance in Tukey test (IL-1β ANOVA *p* < 0.000001, Tukey of respective bar vs. UV, * *p* < 0.0005 and ** *p* < 0.0001, respectively; IL-6 ANOVA *p* < 0.000001, Tukey of respective bar vs. UV, ** *p* < 0.0005; IL-8 ANOVA *p* < 0.000001, Tukey of respective bar vs. UV, * *p* < 0.05 and ** *p* < 0.005).

In the skin, direct sun exposure leads to photo-aging over time and to an inflammatory response called erythema or sunburn immediately after irradiation [12]. Pharmacological topical creams or cosmetic lenitive skincare products are thought to quench or reduce the symptoms of this very common condition. Here, we tested the ability of the extracts to significantly reduce the gene expression of the relevant inflammatory cytokines produced by UV-exposed keratinocytes and fibroblasts, demonstrating their efficiency in counteracting the inflammatory process from the beginning. Indeed, the pro-inflammatory cytokines IL1-β, IL-6 and IL-8, which their expression is known to be significantly induced in the skin by UV irradiation [51], were inhibited in keratinocytes, as revealed by the analysis of their expression profile 6 h after exposure (Figure 7). 

**Figure 8:**
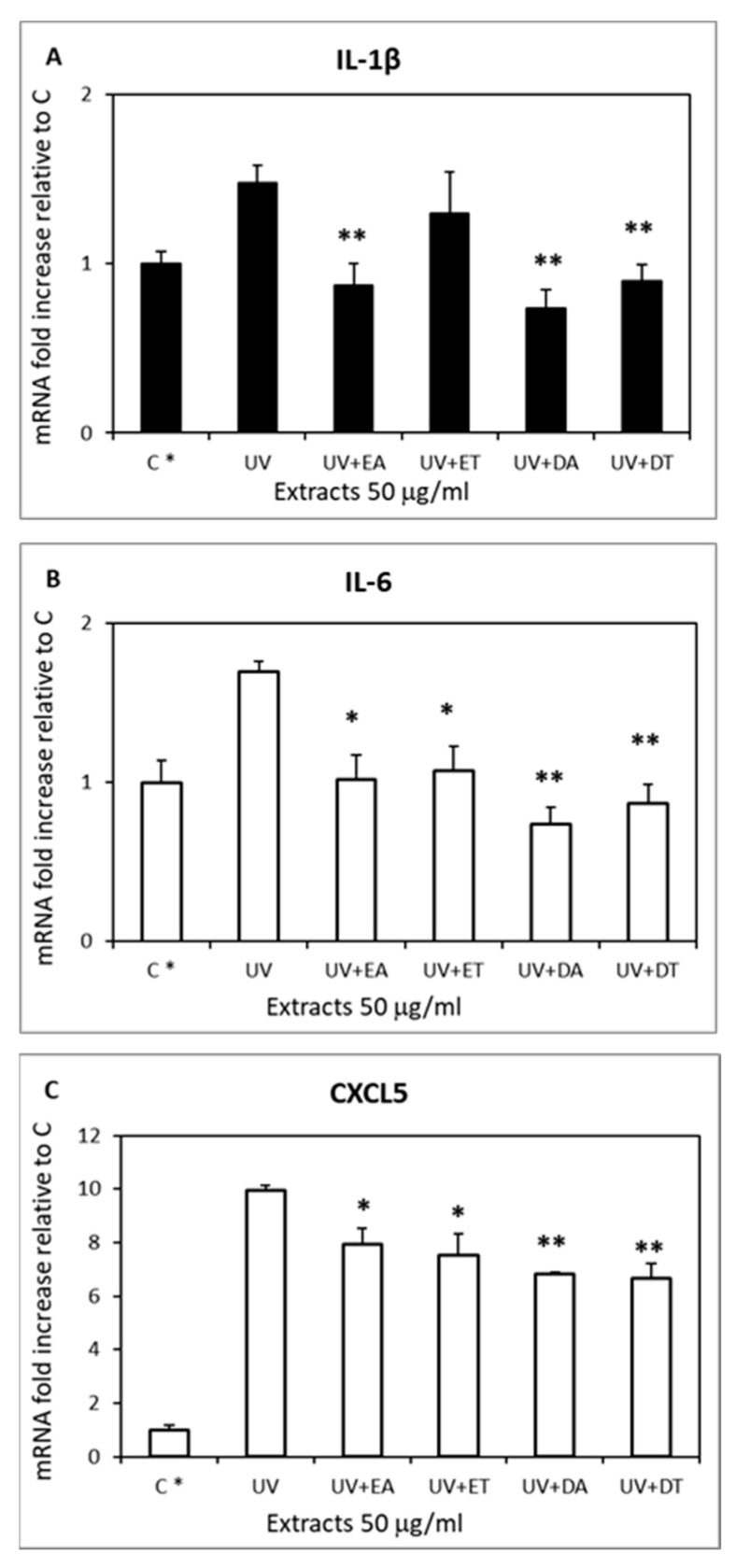
Inhibition of gene expression in UV-challenged L929 murine fibroblasts. Gene expression was measured by qPCR analyses of IL-1β (**A**), IL-6 (**B**), and CXCL5 (**C**) at 6 h, after a 3 min UV challenge and in the presence of 50 μg/mL algal extracts. EA and ET, apices and thallus ethanol extracts; DA and DT, apices and thallus DMSO extracts, respectively. Data are normalized on the GAPDH housekeeping gene and expressed as mRNA-fold increase compared to control, untreated cells. Asterisks indicate significance in the Tukey test (IL-1β ANOVA *p* < 0.0005, Tukey of respective bar vs. UV, ** *p* < 0.005; IL-6 ANOVA *p* < 0.00005, Tukey of respective bar vs. UV, * *p* < 0.005 and ** *p* < 0.0005; CXCL5 ANOVA *p* < 0.000001, Tukey of respective bar vs. UV, * *p* < 0.05 and ** *p* < 0.001).

The four extracts showed different efficacies in inhibiting the three UV-induced cytokines in human keratinocytes. IL1-β was significantly inhibited by all four extracts (Panel A), with the apices EtOH extract showing a slightly higher efficiency (82% inhibition) compared to the others (58–77% inhibition). IL-6 showed a remarkable, comparable inhibition after treatment with both the EtOH and DMSO apices extracts (Panel B, 73% and 77% inhibition, respectively) and a partial but significant inhibition by the thalli DMSO extract. Finally, IL-8’s enhanced gene expression was efficiently inhibited by the apices EtOH extract (78% inhibition) and only partially by the thalli EtOH extract (Panel C). In fibroblasts, the gene expression analysis was performed in the same conditions after UV irradiation on the IL1-β, IL-6 and CXCL5 cytokines (Figure 8). 

The latter was chosen because it is well known that the mouse genome does not contain an IL-8 homologue; therefore, we analyzed the CXCL5 cytokine, which is recognized as the functional homologue of human IL-8 [52], being L929 fibroblasts of murine origin. In this case, the IL1-β UV-induced rise was completely inhibited by the apices EtOH extract and by both DMSO extracts (Panel A), while the IL-6 rise was inhibited by all extracts (Panel B) and CXCL5 was only partially inhibited by all four extracts (Panel C) with a slightly higher efficiency by the two DMSO extracts (35% inhibition). The anti-inflammatory mechanism of action on the two cell types likely depends on the strong ROS-inhibitory effect exerted by the *E. amentacea* extracts. In fact, it is well known that ROSs are one of the main triggers of pro-inflammatory NF-κB transcription factor activation, which in turn, in skin cells, stimulates IL-1β, IL-6, IL-8, TNF-α and MMP1 production, among others [12]. The contribution of meroditerpene molecular species should also be considered since this class of molecules has demonstrated anti-inflammatory properties [53] and because our analyses confirmed a higher content of these compounds in the apices hydroalcoholic extracts (see Section 2.7). Thus, considering the inhibitory effects in the two cell lines of all the three cytokines analyzed, these data indicate that the EtOH extract from the apices displays the highest skin anti-inflammatory properties, confirming our hypothesis that the apical seaweed portion collected during the summer season contains remarkable bioactive molecules that counteract several oxidative and inflammatory symptoms caused by UV radiation and enhance the lenitive effect of those already present in the lower body part (thallus) of the *E. amentacea* seaweed.

### 2.7. Chemical Characterization of E. amentacea Apices and Thalli Extracts

**Figure 9:**
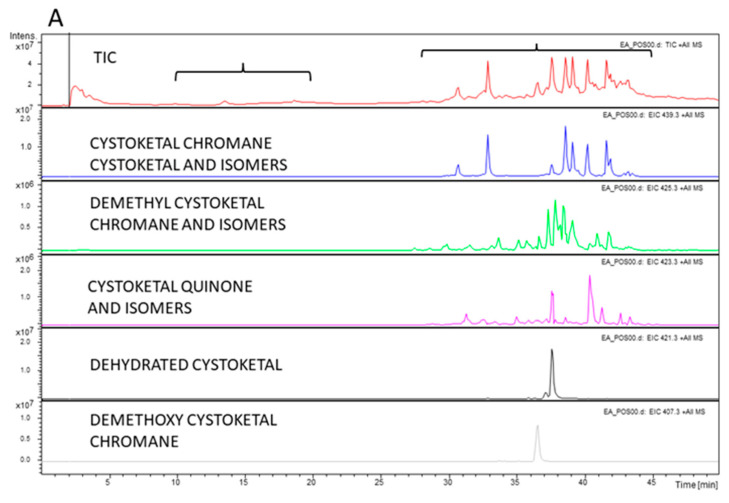

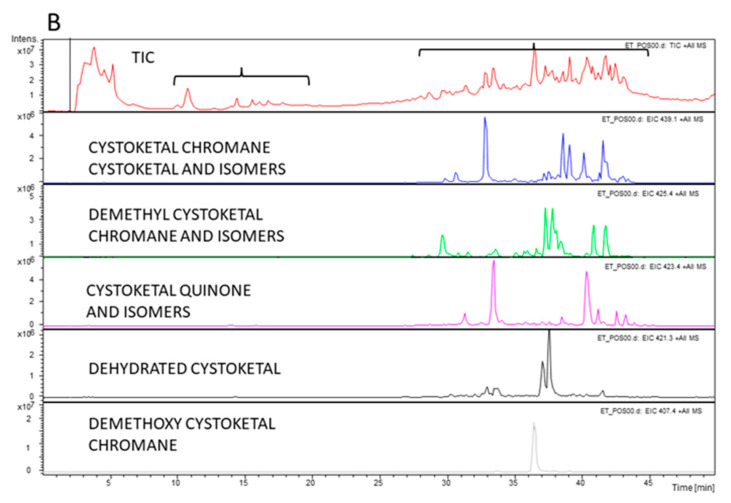
HPLC/MS analyses of the EtOH extracts. Total ion current (TIC) and extracted ion chromatograms (EIC) obtained by full-scan MS/MS analysis coupled to HPLC separation of an aliquot of the *E. amentacea* apices hydroalcoholic (**A**) and thalli (**B**) extracts at a starting dilution of 2.5 mg/mL (injection volume, 8 μL), acquiring the most abundant species under each peak. The red chromatogram in both panels represents the TIC of each extract, while the following blue, green, pink, black and gray chromatograms in both panels represent the EICs of the molecules indicated in the respective panels. The acquisition was performed on negative and positive ions in the 100–1000 mass range and analyzed using integrated software.

As reported in the present work (see the Result paragraphs above), significant functional differences in the biological effects of the extracts were observed, mainly between the apices and thalli hydroalcoholic extracts (EtOH), with the correspondent DMSO extracts showing essentially comparable effects between them. The molecular characterization of the whole thallus of the *E. amentacea* EtOH and DMSO extracts was already obtained in our previous work [23]. Thus, we decided to perform more accurate HPLC-MS/MS analyses on the two apices and thalli EtOH extracts with the aim of identifying the main molecular components responsible for the different biological effects between the extracts of the two algal parts. The analysis is shown in Figure 9, highlighting the molecular species identified in the EtOH apices (Panel A) and in the EtOH thalli (Panel B), respectively. 

In detail, the two MS chromatograms in red (the total ion chromatogram in the first inserts in both Panels A and B) show a group of peaks in the timeframe between a 10- and 20-minute retention time, which the MS analysis ascribed to the polyphenol family, and a second, more significant group of peaks, starting from the 28-minute retention time, belongs to the meroditerpene family. The heights of the peaks in the two chromatograms show that the meroditerpenes species are more abundant in the apices (Panel A) as compared to the thalli (Panel B). Thus, we focused on their characterization to ascribe the functional differences between the extracts from the two body parts of the seaweed. We observed that the two seaweed body parts contained the same meroditerpenes compounds. In fact, both panels reporting the extracted ion chromatograms (EIC) of the same identified molecules show the following species: cystoketal, cystoketal chromane and their related isomers (blue EIC in both panels, *m/z* 439.3), dimethyl cystoketal chromane and their related isomers (green EIC in both panels, *m/z* 425.3), cystoketal quinone and their related isomers (pink EIC in both panels, *m/z* 423.3), dehydrated cystoketal (black EIC in both panels, *m/z* 421.3) and dimethoxy cystoketal chromane (gray EIC in both panels, *m/z* 407.3). Indeed, the presence of meroditerpene-like structures has already been described in the same genus, and some of their biological effects have also been investigated [4,5,36,53,54,55,56,57,58]. Furthermore, the main molecular structures—although without the characterization of the isomers—were also identified in our previous work, both for the EtOH and DMSO extracts of the total fronds of the seaweed [23]. The main chemical structures of the molecules are shown in Table 2, which also reports the literature on the up-to-date investigated pharmacological properties of the listed species.

In our analysis, we can also show that there is a quantitative difference in the abundance of the abovementioned molecules between the apices and thalli, i.e., the younger/reproductive and the slightly older/structural parts of the seaweed fronds, respectively. Table 3 reports a relative quantitative comparison between the abundance of the meroditerpenes species in the two extracts retrievable from the EIC of each molecule. From this analysis, we can formulate the hypothesis that the enhanced biological effects shown by the apices hydroalcoholic extracts, as compared to the thalli, probably are ascribable to the higher amounts of cystoketal, cystoketal chromane and their relative isomers as well as to the dehydrated cystoketal molecules, which are significantly much more present in the apices extracts respectively to the thalli. Future HPLC extraction and purification of these molecules and further analyses of their biological effects will enable the validation of this hypothesis.

## 3. Materials and Methods

### 3.1. Chemicals

All the reagents were acquired from Sigma-Aldrich (Milan, Italy) unless otherwise stated.

### 3.2. Algae Collection

**Figure 10:**
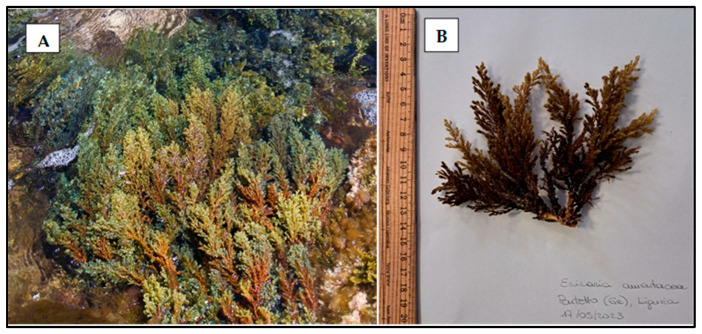
*E. amentacea* specimen. (**A**) Photograph from the site of collection in the Ligurian Sea, in the lower intertidal zone; (**B**) photograph of freshly collected seaweed thalli with a scale bar.

In the Ligurian Sea (Northwestern Mediterranean), fronds of *Ericaria amentacea* were collected in the midlittoral zone, on exposed rocky shores, at Bogliasco, Genoa (NW Italy, 44°22′40.37″ N—9°4′35.14″ E). The collection was performed in July, in the summer of 2020, when the sea temperature values ranged between 25 and 26 °C. After the collection, the fronds were stored in plastic bags, kept in cold conditions, and immediately transported to the laboratory at the University of Genova. No specific permits were required for collecting the specimens. Additionally, non-destructive sampling was performed, as only the upper branches were collected without harming the individual organisms, let alone the whole population. Figure 10 shows the *E. amentacea* photographs from the site of collection in the Ligurian Sea (panel A) and of freshly collected fronds (panel B).

### 3.3. Production of Extracts from E. amentacea

*E. amentacea* fronds were washed with deionized water. The apical parts (with a 3 cm maximum length, referred to in the text as “apices”), distinguishable for the presence of reproductive structures and by a lighter brown color, were cut from the rest of the thallus. Both the apices and thallus fragments (i.e., the primary and secondary branches, devoid of the apical part, referred to in the text as “thalli”; Scheme 1) were air-dried, cut into tiny pieces using a blender and then dried in a lyophilizer. The extracts were obtained by incubation in mild conditions for 48 h in the dark in a rotary disk shaker at 30 °C, as already reported [23]. Briefly, aliquots of 2 g of the lyophilized apices or thalli of the seaweed were extracted in 20 mL of either dimethyl sulfoxide (DMSO) or ethanol: water 1:1 (EtOH). At the end of the incubation, four different extracts were obtained: seaweed apices in DMSO (DA extract) or in EtOH (EA extract), seaweed thalli in DMSO (DT extract) or in EtOH (ET extract). After filtration with a strainer, the aliquots were lyophilized and weighted to determine the yield of the extraction with the two solvents, and finally, the four extracts were diluted to a starting concentration of 25 mg/mL and frozen at −20 °C until further use. The solvent selection was chosen to ensure a higher yield of polyphenol and meroditerpene species extraction since these molecules show remarkable pharmacological activities and seem particularly abundant in the brown algae of the *Ericaria* genre and in this seaweed, as shown in Figure 9 and Table 2. Thus, since these molecular species show both hydrophobic (i.e., phenolic) as well as hydrophilic (i.e., hydroxyl) moieties, the choice of the solvent was a 50% hydroalcoholic solution to attempt to meet the abovementioned compound solubility needs. The DMSO solvent was also chosen because of its broad range of solubility capacity.

### 3.4. Total Phenolic and Flavonoid Contents

The total phenolic content (TPC) was evaluated using the Folin–Ciocalteu assay, as previously reported by Biju et al. [59]. The four *E. amentacea* extracts were quantified at a concentration of 500 µg/mL of the raw extracts, to which the reagents necessary for the TPC quantification were added. The phenolic concentration was obtained by comparison with a calibration curve of gallic acid (from 0.05 to 20 µg/mL final concentrations), and the TPC was expressed as mg of gallic acid equivalents (GAE). 

The total flavonoid content (TFC) was assessed employing the AlCl_3_ colorimetric method, as described by Biju et al. [59]. The four *E. amentacea* extracts were quantified at a concentration of 1 mg/mL of the raw extracts, to which the reagents necessary for the TFC quantification were added. The flavonoid concentration was calculated by comparison with a calibration curve of quercetin (from 1.5 to 30 µg/mL final concentrations), and the TFC was expressed as mg of quercetin equivalents (QE). The TPC and TFC quantifications were performed two times in duplicate.

### 3.5. In Chemico Antioxidant Activity

#### 3.5.1. DPPH Assay

To estimate the total radical scavenging activity of the four *E. amentacea* extracts, the DPPH assay was performed as previously reported [60]. The four *E. amentacea* extracts were tested at 500, 250 and 100 µg/mL final concentrations. Moreover, a negative control with water replacing the extracts and a positive control with ascorbic acid (a 500 µg/mL final concentration) were prepared. The antioxidant activity of the extracts was obtained by estimating the quenching of the DPPH radical with respect to the negative control after subtracting the extracts’ intrinsic absorbance at 517 nm due to their natural color, which was measured in a sample containing only the extracts in water: methanol 1:3. The assay was performed two times in duplicate.

#### 3.5.2. Reducing Fe (III) Power Assay, Hydroxyl Radical and NO Scavenging Activities

The evaluation of the four *E. amentacea* extracts potential to reduce Fe (III) was carried out, as already reported in De La Fuente et al. [23]. The four extracts were tested at 500, 250 and 100 µg/mL final concentrations. Moreover, a negative control with water replacing the extracts and a positive control with ascorbic acid (a 30 µg/mL final concentration) were prepared. Thus, the reducing Fe (III) power of the extracts was calculated as a percentage with respect to the maximum activity of the ascorbic acid positive control. The assay was performed two times in duplicate.

The hydroxyl radical scavenging activity of the extracts was measured employing Mohr’s salt method, according to De La Fuente et al. [23]. The four extracts were tested at 1.5, 1.0 and 0.5 mg/mL final concentrations. In addition, a negative control with water substituting the extracts and a positive control with gallic acid (a 0.5 mg/mL final concentration) were prepared. The test was performed two times in duplicate.

To assess the NO scavenging activity exerted by the extracts, the assay was carried out by De La Fuente et al. [23] by the sodium nitroprusside assay, followed by nitrite quantification through the Griess assay. The four extracts were tested at 500, 250 and 100 µg/mL final concentrations. The NO scavenging activity was calculated as a percentage with respect to a positive control of the sodium nitroprusside solution alone, generating the NO radical by light exposure. For the Griess assay, a calibration curve based on the NaNO_2_ scalar concentrations was used (from 1 to 50 μM). The assay was performed two times in duplicate.

### 3.6. Tyrosinase, Collagenase and Hyaluronidase Inhibition

The tyrosinase inhibition was estimated using the colorimetric assay [61]. An increase in Dopachrome, a highly colored substance obtained by the catalytic conversion of L-Dopa, is proportional to tyrosinase enzymatic activity. Thus, the samples comprised *E. amentacea* extracts (a 100 µg/mL final concentration) in 300 µL of 50 mM Na_2_HPO_4_ (pH 6.8) and 200 µL of L-Dopa solution (25 mM in 50 mM Na_2_HPO_4_, pH 6.8). After 20 µL of mushroom tyrosinase (starting concentration 2500 U/mL) was added to the mixture, the absorbance (ABS) of the sample was immediately recorded at 475 nm for 10 min at 15-second intervals using a Beckman spectrophotometer (DU640). The tyrosinase inhibition was then calculated as a percentage of the activity (activity = speed/min = (ABS at t10′–ABS at t0′)/10) of the positive control (in the presence of the respective vehicle solvents), prepared with 300 µL of 50 mM Na_2_HPO_4_, 200 µL of L-Dopa solution and 20 µL of the enzyme.

The evaluation of the collagenase inhibition was performed according to van Wart and Steinbrink [62]. The inhibition of the collagenase catalytic activity was evidenced by a reduced depletion of FALGPA, a synthetic peptide that mimics the collagen structure. In this enzymatic test, both FALGPA and collagenase were prepared in a reaction buffer made with 50 mM Tricine, 10 mM CaCl_2_ and 400 mM NaCl in deionized water (pH 7.5). After the *E. amentacea* extracts (a 50 µg/mL final concentration) were dissolved in 40 µL of 1 mM FALGPA, 10 µL of collagenase from Clostridium histolyticum (20 U/mL) was added to the sample and its absorbance was immediately recorded at 345 nm for 15 min at intervals of 15 s using a Beckman spectrophotometer (DU640). The collagenase inhibition was obtained by comparison to a positive control (in the presence of the respective vehicle solvents) containing only 40 µL of 1 mM FALGPA and 10 µL of collagenase.

Hyaluronidase activity was assessed using the turbidimetric method described by Bralley et al. [63]. This assay measures the absorbance of the complex between hyaluronan and CTAB (cetyltrimethylammonium bromide) after incubation with the enzyme so that the absorbance is proportional to the remaining amount of undigested hyaluronan. Thus, an absorbance increase is correlated to a hyaluronidase inhibitory effect. For this assay, both hyaluronic acid and hyaluronidase were dissolved in an acetate buffer (0.15 M NaCl in 0.2 M sodium acetate–acetic acid, pH 6.0). Briefly, the test solution comprised *E. amentacea* extracts (50 µg/mL final concentration), 6.6 µL of hyaluronic acid (3 mg/mL), 1.5 µL of hyaluronidase from bovine testes (3000 U/mL) and acetate buffer up to the final volume of 50 µL. The samples were incubated for 15 min at 37 °C before adding 200 µL of the CTAB solution (2.5% CTAB and 2% NaOH in deionized water, pH 12). After 10 more minutes, the optical density was read at 405 nm on an AMR-100 Microplate Reader against a blank with 50 µL of acetate buffer and 200 µL of CTAB. Moreover, since the extracts showed an intrinsic absorbance at 405 nm due to the presence of phenolic compounds, their absorbance values were then subtracted from the value recorded after the enzymatic assay. The hyaluronidase inhibition was obtained by comparison with a positive control containing the enzyme and the reaction substrate without the extracts (in the presence of the respective vehicle solvents). The experiments were performed two times in duplicate.

### 3.7. Cell Cultures

The choice of the mouse fibroblast L929 and the human keratinocyte HaCaT cell lines was made since these are widely used biological systems in a very high number of cytotoxicity as well as cosmetic studies [64,65,66,67]. Concerning the L929 non-human fibroblasts, they were used because of the reproducibility of the past results obtained in several studies from our lab for cosmetic/pharmacological purposes [23,60,68,69,70] and because the use of the same cell line allowed for comparisons with previous results using similar extracts of the same seaweed [23].

The mouse fibroblast L929 cell line and the human keratinocyte HaCaT cell line used in this study were obtained, respectively, from the American Type Culture Collection (LGC Standards srl, Milan, Italy) and the Cell Lines Service (CLS GmbH, Eppelheim, Germany). Both cell lines were cultured at 37 °C in a humidified 5% CO_2_ atmosphere in high glucose DMEM with 2 mM L-glutamine (Euroclone, Milan, Italy), supplemented with 10% FBS (Euroclone) and penicillin/streptomycin as the antibiotics (Corning Inc, Corning, NY, USA).

### 3.8. Cell Toxicity and Rescue from UV and H_2_O_2_ Treatment

The potential cytotoxicity of the *E. amentacea* extracts was estimated in both the L929 fibroblasts and HaCaT keratinocytes. In fact, after the cells were seeded in 96-well plates at a density of 10,000 cells/well and allowed to adhere overnight, they were treated with different extracts (100, 50 and 10 µg/mL final concentrations). The plates were incubated for 24 h at 37 °C, and at the end of the experiments, the cell viability was measured with the MTT assay, as previously reported in Pozzolini et al. [68]. The results are the means ± SD of three independent experiments, in which each condition was tested eight times, including the solvents alone (1% final dilutions).

The extracts’ efficiency in reducing the H_2_O_2_- or the UV-induced cytotoxicity was assessed in the L929 fibroblasts and HaCaT keratinocytes, which were plated in 96-well plates at 10,000 cells/well and allowed to adhere overnight in a complete medium. The cells were then alternatively challenged with H_2_O_2_ 200 µM or irradiated for 2 or 5 min under a UV lamp (Sanikyo Denki G20T10) at a 20 cm distance (90 mJ/cm^2^ and 227 mJ/cm^2^ total radiation doses, respectively), in the presence or absence of the *E. amentacea* extracts (100, 50 and 10 µg/mL concentrations). After 24 h of incubation, the cell viability was assayed by the MTT test and compared to the control—the untreated cells. Data are the means ± SD of three independent experiments in which each condition was tested eight times. Preliminary experiments were previously performed on these two cell lines to establish the suitable radiation doses that were able to induce significant cell death while still allowing us to measure the eventual rescue of cell viability, both mimicking a milder rate and a more severe exposure rate (90 mJ/cm^2^ and 227 mJ/cm^2^, respectively). These radiation values were based on the previous papers [71,72] mainly applied in the in vivo studies. Furthermore, the same conditions used in this work are already reported in our previous study, where the same cell lines and experimental settings were satisfactorily used [68]. 

### 3.9. Inhibition of Intracellular ROS Production

L929 fibroblasts and HaCaT keratinocytes were seeded in 96-well plates at a density of 10,000 cells/well, and on the following day, the assay was performed according to Pozzolini et al. [73]. Briefly, after being washed once with Hank’s Balanced Salt Solution (HBSS), the cells were incubated for 45 min at 37 °C with 10 µM 2′,7′-dichlorodihydrofluorescein diacetate dye in HBSS (Life Technologies). Once washed with HBSS to remove the excess dye, the cells were either UV-irradiated for 3 min or challenged with 200 µM H_2_O_2_ and then treated with the *E. amentacea* extracts (the 100 µg/mL or 50 µg/mL final concentrations). Following 2 h of incubation, the plates were read on the Fluostar Optima BMG at 485/520 excitation/emission wavelengths. Data are the means ± SD of three independent experiments in which each condition was tested eight times.

### 3.10. Gene Expression Analyses

After being seeded in the 6-well plates at 300,000 cells/well and allowed to adhere overnight, the L929 fibroblasts were UV-irradiated for 5 min and treated with 50 µg/mL of the *E. amentacea* extracts. The gene expressions of interleukin-1β (IL-1β), interleukin-6 (IL-6) and chemokine (C-X-C motif) ligand 5 (CXCL5) were quantified by qPCR after 6 h. Alternatively, the HaCaT keratinocytes, seeded on the previous day in 6-well plates at a density of 300,000 cells/well, were UV-irradiated for 2 min and treated with 50 µg/mL of the extracts. The gene expressions of interleukin-1β (IL-1β), interleukin-6 (IL-6) and interleukin-8 (IL-8) were measured by qPCR after 6 h. At the end of the incubation, the RNA was extracted using the NucleoSpin RNA Mini kit (MACHEREY-NAGEL, Dueren, Germany) and subsequently analyzed using a NanoDrop spectrophotometer (Nanodrop Technologies, Wilmington, DE, USA). The cDNA was synthesized from 1 µg RNA using the iScript cDNA Synthesis Kit (Bio-Rad Laboratories, Milan, Italy). The PCR reaction (10 µL volume) contained: 4 × master mixes (Biotechrabbit GmbH, Hennigsdorf, Germany), 0.2 µM primers and 5 ng of cDNA. The analysis was performed in triplicate for each sample. The thermal conditions were: 3 min at 95 °C for the initial denaturation, followed by 45 cycles with 15 s at 95 °C for denaturation and 60 s at 60 °C for annealing and elongation. The samples were normalized on GAPDH and HPRT-1 (housekeeping gene) mRNAs for the L929 fibroblasts and HaCaT keratinocytes, respectively. The primer pair design (Table 4) was obtained using the Beacon Designer 7.0 software (Premier Biosoft International, Palo Alto, CA, USA) and synthesized by TibMolBiol (Genova, Italy). Data analyses were obtained using the DNA Engine Opticon 3 Real-Time Detection System Software program (3.03 version, Bio-Rad, Milan, Italy). The experiments were performed two times in triplicate.

### 3.11. HPLC/MS Analyses

The chromatographic separation of the two extracts was carried out using the Agilent 1100 µHPLC equipped with an automatic micro-sampler and an XSelect C18 column (300 Å pore size, 5 µm particle size, 1 mm, internal diameter, ×150 mm, length, respectively) maintained at 30 °C. The injection volume was 8 µL (with a starting concentration of 2.5 mg/mL). The chromatographic method comprised the following gradient of 45 min: 0–5 min 8% B, 5–40 min from 8% to 100% B, 40–45 min 100% B, at a flow rate of 30 µL/min, where A was H_2_O containing 0,1% formic acid (FOA) and B was acetonitrile 0.05% FOA. The detector was set at 220/280 nm. Finally, the HPLC was coupled with the mass spectrometer (HPLC-ESI-MS) to qualitatively evaluate the compounds in the extract.

The instrument used was a mass spectrometer with an electrospray ion source (ESI) and a high-capacity ion trap (Agilent 1100 MSD XCT ion trap). All parameters were established to obtain the best ionization of the components. The analysis was performed in an ion-charge mode control with a target selected at 100,000 and an accumulation time of 300 ms. The operating parameters were as follows: capillary voltage, 3.6 V; nebulizer pressure, 20 psi; drying gas, 10 L/min; dry temperature, 350 °C; moving averages, 3; fragmentation width, 1 V, respectively. All mass spectra were acquired in full-scan and MS-MS mode, acquiring the most abundant species under each peak. The acquisition was performed on negative and positive ions in the 100–1000 mass range and analyzed using the integrated Agilent Data Analysis software (LC/MSD Trap software 5.3).

### 3.12. Statistical Analyses

Statistical analyses were performed using one-way ANOVA plus Tukey’s post-test (GraphPad Prism Software 5.0, Inc., San Diego, CA, USA). The significance level of the tests was set at 0.05.

## 4. Conclusions

The data obtained in this study demonstrate the remarkable photoprotective and anti-aging effects of the hydroalcoholic and DMSO extracts from two portions of the Mediterranean seaweed *E. amentacea* (i.e., algal apices and thalli) by molecular and cellular analyses, highlighting the significantly stronger beneficial effects from the extracts of the apical portions. We were able to demonstrate the postulate that because during summer, the apical portions grow and hold reproductive structures, and given that in this season, the algae are subjected to the highest solar UV radiation, these upper seaweed portions would be particularly enriched in antioxidant and photoprotective molecules which can counteract the detrimental effects of UV-B radiation on the cellular skin models. In fact, although all the extracts were enriched in polyphenols, flavonoids and ROS scavenging molecules and showed significant biological activities, the apices extracts, and those retrieved by hydroalcoholic extraction, demonstrated the highest pharmacological potential, likely due to the abundance of meroditerpenes structures, such as cystoketal, dehydrated cystoketal, cystoketal chromane and some of their related isomers. The extracts showed cell rescue, antioxidant and anti-inflammatory abilities in the UV-exposed keratinocyte and fibroblast cellular models. They ultimately restored cell viability in both cell types, abated the intracellular oxidative stress and significantly reduced the production of important mediators that propagate and exacerbate the inflammatory process, i.e., IL-1β, IL-6 and IL-8, which are typically released after sunburns and contribute to erythema formation and pain. Furthermore, the algal extracts showed important in vitro hydrolytic skin enzyme inhibition abilities by significantly reducing collagenase and hyaluronidase degrading activities and anti-sunspot formation properties by partially blocking the tyrosinase enzymatic activity. Thus, a good biocompatibility and cell growth-promoting effect in the skin in the in vitro models was clearly demonstrated, alongside remarkable multifactorial anti-aging properties, which were particularly concentrated in the apical portions of the seaweed. Therefore, the *E. amentacea* algal extracts may constitute ideal additives to cosmetic and/or phyto-pharmaceutical products for lenitive and photoprotective treatments to cure UV-derived skin inflammatory states and to promote skin renovation in elders.

## Figures and Tables

**Table 1:** TPC and TFC of *E. amentacea* extracts.

Extract	TPC (μg/mg extr)	TFC (μg/mg extr)
EtOH Apex	48.32 ± 0.54	5.60 ± 0.66
EtOH Thallus	18.47 ± 3.49	2.28 ± 0.27
DMSO Apex	39.40 ± 1.94	2.433 ± 0.35
DMSO Thallus	55.52 ± 6.08	4.45 ± 0.19

Quantification of the total phenolic content (TPC) and total flavonoid content (TFC) by the Folin–Ciocalteu and AlCl3 colorimetric assays, respectively.

**Table 2:** Chemical structure of the identified compounds. The main meroditerpenes molecules identified in *E. amentacea* apices and thalli hydroalcoholic extracts and references related to reported biological activities.

Molecular Structure	Name	References
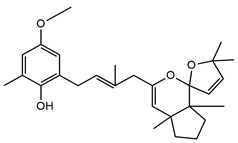	Cystoketal	[56,57]
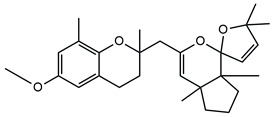	Cystoketal Chromane	
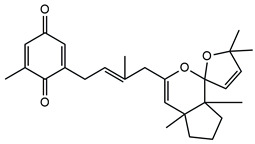	Cystoketal quinone	[58]
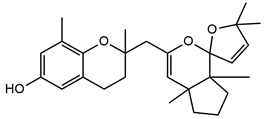	Demethyl cystoketal chromane	
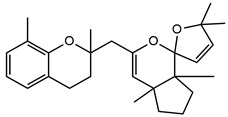	Demethoxy cystoketal chromane	[4,36]
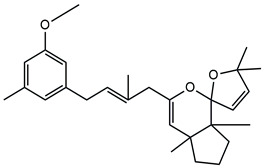	Dehydrated cystoketal	

**Table 3:** Compounds’ relative abundance in the apices and thalli hydroalcoholic (EtOH) extracts.

Identified Compounds	Abundance in Apices	Abundance in Thalli
Cystoketal, cystoketal chromane and isomers	++++	+
Demethyl cystoketal chromane, and isomers	+	+++
Cystoketal quinone, and isomers	+	+++
Dehydrated cystoketal	++++	+
Demethoxy cystoketal chromane	+	++

Indication of the relative abundance of the main meroditerpenes identified in the apices and thalli hydroalcoholic (EtOH) extracts obtained by the comparative analysis of the respective extracted ion chromatogram for each molecule in the two extracts. The plus signs, from + to ++++, indicate an increasing degree of abundance of the respective molecule.

**Table 4:** List of primers used in qPCR experiments.

GENE	GenBank (a.n.)	Forward	Reverse	Size (bp)
**Human:**				
IL-1β	NM_008361.4	gCAgCACATCAACAAgAg	CAgCAggTTATCATCATCAT C	184
IL-6	NM_031168.2	ACCTgTCTATACCACTTC	gCATCATCgTTgTTCATA	117
IL-8	NM_000584.4	AATTCATTCTCTgTggTATC	CCAGGAATCTTgTATTgC	127
HPRT-1	NM_000194.3	ggTCAggCAgTATAATCCAAAg	TTCATTATAgTCAAgggCATATCC	144
**Murine:**				
CXCL5	NM_009141.3	TgCTTAACCgTAACTCCAA	ATCCAgACAgACCTCCTT	129
IL-1b	NM_008361.4	gCAgCACATCAACAAgAg	CAgCAggTTATCATCATCATC	184
IL-6	NM_031168.2	ACCTgTCTATACCACTTC	gCATCATCgTTgTTCATA	117
GAPDH	NM_001289726.1	TCTCCCTCACAATTTCCATCCCAg	gggTgCAGCgAACTT TATTgATgg	99

## Data Availability

Data sharing is not applicable to this article.
